# An Innovative Resource to Guide and Track Medical Resident Activity and Wellness During At-Home Assignments

**DOI:** 10.7759/cureus.40332

**Published:** 2023-06-12

**Authors:** Diane M Jarrett, Lauren Gibson-Oliver, Shashank Kraleti

**Affiliations:** 1 Department of Family and Preventive Medicine, University of Arkansas for Medical Sciences, Little Rock, USA

**Keywords:** documenting work, medical resident education, work from home, parental leave, residency program resources

## Abstract

Background and objectives

Medical residents may work from home for various reasons, including study electives, isolation due to exposure to illness, or during parental leave when they choose to work at home instead of taking extended time off. The University of Arkansas for Medical Sciences (UAMS) Little Rock Family Medicine residency program recognized the need for a tool that provided residents with a list of resources and approved activities for at-home work and a means of tracking their performance in those activities.

Methods

The administration team at the UAMS Little Rock Family Medicine residency program custom-designed a Daily Activities Log that served multiple purposes. Family medicine residents used it to choose what activities to participate in from a comprehensive list of activities and resources including virtual conferences, recorded didactics, modules, and other online materials. The program provided the framework on the log, while residents used it to document time spent on those activities. The log also gave the program a daily update on the resident’s health, as one question specifically asked about well-being. Since it was built in an electronic survey format already owned by the residency program, it was completely customizable and available at no additional cost.

Results

In the two years covered by this project, residents logged a total of 593 hours of at-home work. In response to a survey, 76% of participating residents (N=14) rated the log as extremely or very easy to use; 64% indicated that it was a helpful resource; and 50% said that it simplified the reporting of their daily status. The residency program faculty found that having one source to track all off-site residents was an efficient means of monitoring their well-being and their work.

Conclusions

The Daily Activities Log is a versatile tool that provides comprehensive information, resources, and approved activities for residents, documents their work, and provides updates to residency management. It can be readily modified for use in other programs that have residents working at home.

## Introduction

Working from home at least part of the time has become an increasingly popular option for employees in many fields. In the United States between 2019 and 2021, the number of people working mainly from their homes tripled [1]. The concurrent COVID-19 pandemic supported the growth of at-home work, both to slow the spread of infection and to accommodate employees who were well enough to work but not well enough to report to the office [2,3].

Those in medical residency training may have several reasons for needing options to study or work from home for a portion of their program. They obviously were at particular risk during the pandemic, leading to increased virtual training to ensure their safety and health [4-6]. COVID-19 also landed some residents in quarantine even though they were asymptomatic and able to participate in remote activities [7]. They needed options to remain engaged while working from home.

Other reasons for at-home assignments can include study electives for residents who are preparing for exams and who need dedicated, intensive study time. These exams include the Step 3 exam from the United States Medical Licensing Examination (USMLE) and the Level 3 COMLEX exam (Comprehensive Osteopathic Medical Licensing Examination for students who graduated from an osteopathic medical school) [8,9]. Another exam that may require intensive study is the board certification exam taken shortly before graduation from the residency program. For family medicine residents, that exam is produced by the American Board of Family Medicine (ABFM) [10].

In addition, some residents on parental leave after the birth or adoption of a child want the flexibility to work from home after their formal leave period has ended [11-13]. This extra time at home is supportive of resident wellness [14], child development [15], and breastfeeding, which is particularly challenging for residents during a short parental leave [12,16]. A 2019 study found that 73.4% of responding family medicine programs offered at-home elective options for new parents [17].

With the increasing numbers of residents asking for at-home assignments, residency programs are tasked with identifying suitable activities and work that can be performed at home; instructing the residents about the activities; and finding a means of documenting the work that residents are involved with when not in the office. In response to increased requests for at-home assignments, the UAMS Little Rock Family Medicine residency program faculty began searching for available resources and also devising tools of their own to keep their off-site residents engaged and productive.

## Materials and methods

Study population

The University of Arkansas for Medical Sciences (UAMS) Institutional Review Board approved this study as exempt (study #274311). The UAMS Little Rock Family Medicine residency program is a 6-6-6 (three years, with six residents per year) program located at the UAMS Medical Center in the state’s capital.

Initial tracking of at-home learning processes and engagement

Since the onset of the COVID-19 pandemic in 2020, several residents in the Family Medicine program were placed on quarantine after testing positive for the virus. Others were isolated pending test results. Regardless of the reason, some residents felt well enough to work remotely and needed direction on the tasks they could perform that would keep them productive as they waited for clearance to return to the office.

The residency administrative team soon recognized the need for an innovative tracking system. To keep residents engaged, the team needed to specify structured learning opportunities for residents to experience during their absence. The numbers of affected residents and their varying situations were too complex for the existing policies and forms. Additionally, no system was in place for residents working remotely to document their time, and no framework of suitable activities was readily available for them to use in planning each day they were out of the office.

Expansion of project to non-COVID activities (e.g., study electives, parental leave)

The residency administrative team designed a Daily Activities Log in response to the COVID-19 situation, but it quickly became obvious that the log could be used for study electives or other at-home assignments. It provided a comprehensive description of expectations and approved activities for residents working on at-home assignments in general, including residents on study electives, or in isolation due to exposure or illness. Additionally, it provided a means for residents to document the time they spent on each activity. Since one question asked specifically about resident wellness, it also served as a wellness check.

The log could also be used for residents on parental leave. Though the accrediting body allots six paid weeks off for medical, parental, and caregiver leave, some residents requested to work from home during part of their leave or in addition to their leave. They needed the framework to know what resources were available and to document their time and activities.

The activities log was set up with a web link to an electronic survey that is accessible from any device. Since the program had already purchased an electronic survey platform for other purposes, they could work within the same platform to custom-design and launch the Daily Activities Log at no additional cost to the program.

The process of the Daily Activities log

Residents who plan to be working at home on a given day document their status on the Daily Activities Log, beginning with a question about their wellness on that day. Then they log the activities they participated in, drawing from the log's comprehensive list of approved activities and resources, and the number of hours devoted to each. The log is available from a web-based link that can be used for an unlimited number of days.

## Results

Use of the Daily Activities Log

Over the two years covered by this project, 17 family medicine residents logged a total of 95 days and 593 hours of activities that were generally out of office and not part of the usual routine. Table 1 shows the activities that were logged by the residents. In 2020-2021 during a pandemic surge, several healthy residents were assigned to COVID-19 triage. They used the log to keep track of their time away from their usual training (See "Triage" in Table 1). Other activities logged by the residents over the two years included EMR (electronic medical record) and administrative tasks, virtual conferences, and presentation preparation.

**Table 1:** Daily Activities Logged by At-Home Residents, 2020-2022 *“Other” activities included completing paperwork, EMR training, meetings with faculty, orientation videos, and preparing for upcoming patient encounters.

ACTIVITIES	2020-2021 (hours)	2021-2022 (hours)	SUBTOTAL BY ACTIVITY (hours)
AAFP (American Academy of Family Physicians) question banks	7.5	10.5	18
Administrative tasks	10	36.5	46.5
Behavioral Health Integration modules	0.5	0	0.5
EMR (electronic medical record) tasks	27.5	21	48.5
FMC (Family Medical Center) shadowing	0	12	12
IHI (Institute for Healthcare Improvement) modules	18.5	0	18.5
Journal Club preparation	0	3.5	3.5
Journal reading	30	6	36
Noon virtual conference	16	12	28
PPFM (Practical Problems in Family Medicine) presentation preparation	0	2	2
QI (quality improvement) project	0	4.5	4.5
RCR (Residency Curriculum Resource)	4	0	4
Readings required for a rotation	0	8.5	8.5
Rosh Review test preparation	22	0	22
SonoSim online training	33.5	0	33.5
Teleconferences from Arkansas Children’s Hospital	1	0.5	1.5
Test preparation	0	24.5	24.5
Triage	111.5	0	111.5
Virtual conferences	7.5	9	16.5
Wellness activities	24	16	40
Other*	61.5	51.5	113
SUBTOTALS	375	218	n/a
TOTAL: 593 hours logged over 2 years			

Table 2 shows actual examples of resident workdays by post-graduate year (PGY). A PGY1 resident is in the first year of the residency program, while PGY2 and PGY3 residents are in the second and third years, respectively. On the Daily Activities Log, residents checked off the activities they had participated in and indicated the number of hours spent on each task. Faculty members who were monitoring the off-site residents could see at a glance what activities the residents participated in and how many hours they worked on a daily basis.

**Table 2:** Examples of Workdays Documented by Residents on At-Home Assignments

ACTIVITIES CHOSEN FROM THE CHECKLIST ON THE DAILY ACTIVITIES LOG	PGY3 example day	PGY2 example day	PGY1 example day
Administrative tasks	3.5 hours	2.5 hours	0
EMR (electronic medical record) tasks	0	0	30 minutes
Journal Club preparation	1.5 hours	0	0
QI (quality improvement) project preparation	0	1 hour	0
Readings required for a rotation	30 minutes	0	2 hours
Telemedicine patients	0	4 hours	0
Test preparation	1.5 hours	0	4 hours
Virtual conference	1 hour	0	1 hour
Virtual meeting	0	30 minutes	0
Wellness activities	0	0	30 minutes

Evaluating the Daily Activities Log

To elicit resident opinions on the use of the Daily Activities Log, an electronic survey was designed and distributed by the residency program's director of education. The survey introduction indicated that it was confidential, with no tracking information available, and that resident input would be used to improve the overall process of coordinating and monitoring activity outside of the office. There were 17 residents who completed the log over the two years. Two of them had graduated and were excluded from the survey. Of the remaining 15 residents, 14 responded to the survey.

Table 3 shows the resident response to a survey about their opinions of the Daily Activities Log. Half of the residents (7 out of 14) indicated that the log was either extremely or very helpful in reporting their daily status, and 64% (9 out of 14) reported that as a resource for activities, it was either extremely or very helpful. The top rating was for ease of use, which was 76% (11 out of 14 rating it as extremely or very helpful). At least two of the respondents seemed to lack strong memories about using the log as can be noted by their responses and comments, but this may have been because they had not seen or used the log in almost two years.

**Table 3:** Resident Evaluations of the Daily Activities Log, With Comments

Rating	Not much	Somewhat	Very	Extremely
Easy to use	0	1	6	5
Helpful resource	1	2	3	6
Ease of reporting daily status	1	2	1	6
COMMENTS				
It gave me the opportunity to continue to learn, work, and participate while away from work.				
I did not take any time out of the clinic/office due to COVID-19 during my residency training.				
Would have been beneficial to have known about ABFM requirements as part of Activities Log.				
It was very useful.				
Recommend adding "UWorld practice questions" to the list as many residents use this for board prep.				
The Daily Activities Log was easy to use and also provided a list with the options to plan and schedule the activities for the week.				
It was a good tool.				
It was easy and helpful to communicate with the entire administrative team instead of sending emails or via phone call. The resources were helpful and served as a reminder for planning on doing pending work.				
Easy to use.				
I don’t recall this at all or even remember it being mentioned as an option.				
[I spent] at least five hours on [an online training module]. It helped me comprehend shoulder anatomy a lot better, a topic I have struggled with since med school.				
Great.				
I believe the daily activities log was easy to fill out. I'm not sure if I remember the log activities, but I remembered there were times where I had something related to residency but that specific item was not listed for me to select. I would need to see the list again to be more specific.				

## Discussion

Despite the abatement of the COVID-19 pandemic, residency programs still face a variety of reasons as to why residents need options for at-home work and study. Parental leave is a particularly important consideration, as residents often become parents while in training [12,18,19]. Extra time at home after formal parental leave has ended can provide residents with much-needed support during what can be a very stressful time during their training [20].

Other options may include at-home work to complete research and quality improvement projects [7] and to study for exams. A 2022 qualitative study found that a “reserve” rotation with an independent study curriculum yielded unexpected benefits as indicated by a resident who commented, “We worked less than we normally did and I had time to catch up on some projects that I didn’t necessarily have time for before. I got to actually study for boards" [2].

Regardless of the reason, at-home work options provide residents with the opportunity to decrease interruptions and extensions in training. Family medicine residency programs, for example, are typically 36 months. One possible consequence of protracted leave time is the extension of the residency program to enable the residents to make up for the training they missed. Working at home (as opposed to extended leave) can support the resident in graduating on time and being available for opportunities such as fellowships that often begin immediately after the regular end-of-residency date [12].

Though at-home training options have become more common, we found few specifics on how a program can provide at-home residents with comprehensive information on approved activities and the framework for documenting those activities all in one format. Ben-Zion et al. (2022) reported that residency programs often provide a list of activities from which to choose during a parenting elective but added that specific information on how such electives are organized is difficult to find [11]. Cree-Green et al. (2020) discussed how they developed a structured parenting elective that included reading articles, preparing a presentation, etc. They noted that they loaded elective materials on an electronic tablet for each resident but did not discuss how residents were monitored or how their work was documented during their time at home [12].

We discovered that one easy-to-use tool can serve both purposes, in addition to giving the program a simple means of monitoring resident well-being when they are away from the training site. Faculty members and administrative staff found that the log gave them a quick overview of resident performance even at a time when they were at a distance from the office and the clinic. Residents indicated their satisfaction with having a tool that provided clear expectations about their offsite assignments.

By designing our own log as opposed to attempting to use commercial products or apps, we were able to customize the content completely and make changes as often as necessary. Since the log was built as part of a survey platform that we had already purchased, there was no additional cost to the program.

Based on our success, we believe the Daily Activities Log can be adapted for use in a variety of residency programs with a need to monitor residents who are assigned to at-home work. Its ability to be customized continually to the evolving needs of a specific program make it an essential element of residency training in an off-site setting.

## Conclusions

Though this study is limited by the small sample size (17 residents at one family medicine residency program), it provides an innovative means of custom-designing an activities log that offers both a list of approved activities and the means to document participation all in one easy-to-use, inexpensive survey format.

Other limitations include the possibility of social-desirability bias or other response biases that might lead respondents to answer questions in an effort, conscious or not, to make themselves look good. Another limitation is that while some of the daily activities (e.g., online resources with time stamps) could be verified, others relied upon the honesty of the respondents to report what they were doing outside of the office. Additional research is necessary to address these issues.

Future areas for study include identifying additional approved activities and expanding the use of the Daily Activities Log to include tracking of rotational assignments, required readings, and didactics attendance. Various tracking resources exist for such activities but not necessarily all in one framework that is adaptable to multiple uses. Other resources and apps also may involve fees, whereas our log did not. Another promising possibility is requiring residents to produce scholarly work based on their offsite training and to log that work in this same format, one that includes data from the beginning of training through eventual scholarly output.

As more medical residents request the option of at-home work, residency programs must be ready to provide a functional framework to support them. A flexible, no-cost, customizable Daily Activities Log proved its merit in this study. Other programs and groups can use this format as a basis to custom-design surveys to meet their own needs.
